# Use of a Photocleavable Initiator to Characterize Polymer Chains Grafted onto a Metal Plate with the Grafting-from Method

**DOI:** 10.3390/polym15051265

**Published:** 2023-03-02

**Authors:** Flavien Mouillard, Tom Ferté, Emilie Voirin, Stéphane Méry, Patrick Masson, Adele Carradò

**Affiliations:** Institut de Physique et Chimie des Matériaux de Strasbourg, IPCMS, UMR 7504 CNRS, Université de Strasbourg, 67000 Strasbourg, France

**Keywords:** surface-initiated polymerization, ”grafting-from” method, selective cleavage, analysis in solution, ATRP, polymer brushes

## Abstract

The thorough characterization of polymer chains grafted through a “grafting-from” process onto substrates based on the determination of number (Mn) and weight (Mw) average molar masses, as well as dispersity (Ɖ), is quite challenging. It requires the cleavage of grafted chains selectively at the polymer–substrate bond without polymer degradation to allow their analysis in solution with steric exclusion chromatography, in particular. The study herein describes a technique for the selective cleavage of PMMA grafted onto titanium substrate (Ti-PMMA) using an anchoring molecule that combines an atom transfer radical polymerization (ATRP) initiator and a UV-cleavable moiety. This technique allows the demonstration of the efficiency of the ATRP of PMMA on titanium substrates and verification that the chains were grown homogeneously.

## 1. Introduction

There are composite materials in nature that are built of biopolymers connected by chemical groups, such as carbonates or phosphonates, on inorganic cores, including silica and silicates [1]. In this regard, investigations based on these natural interactions have been conducted. Mutin et al., for instance, functionalized metal oxides with organophosphorus acids and their derivatives (salts, esters, etc.) using both the sol-gel technique and surface treatment [2]. They demonstrated that phosphorus atoms permitted Ti–O–P coordination bonds to form between metal substrates and organic molecules. In another study, Pham et al. worked on hybrid composites based on silver (Ag) NPs and graphene oxide bearing thiol groups. After synthesizing the thiol groups from graphene oxide and phosphorus pentasulfide, the Ag NPs were linked to the thiol groups. Specifically, the presence of sulfur atoms caused the formation of coordination bonds between thiol groups and metal nanoparticles [3]. Farkaš et al. explored cobalt nanoparticles combined with carboxylic acids more recently. They demonstrated that carboxyl groups permitted covalent bond formation between inorganic substrates and organic molecules [4]. Particularly, the inclusion of a phosphorus atom capable of interacting with inorganic materials and organic molecules is an intriguing strategy that facilitates the development of stable Ti–O–P bonds [5].

Based on these studies, surface modification using polymer brushes has become a powerful approach to adapt the physical and chemical properties of materials for specific applications. More particularly, surface-initiated controlled radical polymerization (SI-CRP) has been widely studied [6]. This approach consists of growing polymer chains on surfaces to form thin layers, one end of which is linked to a solid interface [7]. It allows the control of thickness of a polymer interface, which makes it usable in many applications, such as catalysis, electronics [8], nanomaterial synthesis [9], and biodetection [10], as well as for the functionalization of substrates such as natural fibers, polymer nanofibers, mesoporous materials, graphene, and proteins [11]. The polymerization mechanisms of SI-CRP are identical to those of CRP in solution, except that this reaction takes place in a dense medium, more precisely, at the interface of the solid and liquid phases [12]. The characterization of these polymer brushes’ growth on metallic surfaces constitutes a challenge, especially for determining specific polymer properties, such as grafting density, molecular weight, and dispersity (Ð). Actually, it is rather difficult to study these types of properties without cleaving the metal–polymer strong bonds. There are several ways to separate grafted polymer chains from a substrate, such as using physical routes, chemical reagents, or mechanical techniques. Dissolving the metal support in a corrosive liquid, such as a strong acid or base, is the easiest method for removing a grafted polymer from its metal support. This corrosive medium can also break the binding between these two components by forming new chemical bonds that are more stable than those that hold the polymer and metal together. In the case of iron oxide nanoparticles (NPs) coated with polystyrene (PS) or poly(methyl methacrylate) (PMMA), for instance, a combination of tetrahydrofuran (THF) and hydrochloric acid dissolves the NPs and permits the extraction of the free polymer [13]. Hydrofluoric (HF) acid is used to cleave PS, PMMA, polyacrylonitriles, and even poly(2-dimethylamine ethyl methacrylate) from silica nanoparticles coated with polymers. Polyethylene (PE) chains have been severed from silica nanoparticles using potassium hydroxide. Koylu et al. [14] investigated the breakage of acid-sensitive chains in response to a polymer’s chemical groups. To achieve this, *p*-toluene sulphonic acid was employed to collect PS chains that were grafted onto silicon (Si) wafers. However, these chemical methods are only compatible with polymers that are resistant to acidic and corrosive conditions.

Earlier studies have shown that it is possible to use the “grafting-from” method [15] to design a novel hybrid material for biomedical purposes. More particularly, Reggente et al. used surface-initiated atom transfer radical polymerization (SI-ATRP) to grow PMMA chains on Ti plates using a three-step strategy [16]. After mechanical polishing of the surfaces, Ti plates were activated by immersion in a sodium hydroxide (NaOH) solution that favored the presence of hydroxyl groups (–OH) on the metallic surfaces. Subsequently, these chemical groups allowed the grafting of a polymerization initiator, bromoisobutyrate-undecenylphosphonic acid (C_15_H_30_O_5_PBr noted C11). The phosphonic acid function at one end of this initiator reacted with the –OH functions of the Ti surface to form Ti–O–P strong bonds. Finally, the α-bromoester present at the outer end of this initiator allowed the polymerization of MMA by SI-ATRP directly on the metal substrate. This “grafting-from” approach enabled the production of dense, micrometer-thick PMMA layers grafted with strong bonds on the Ti surfaces. In order to characterize the polymer in solution, the Ti-PMMA bonds were tentatively cleaved in response to these studies, similar to the procedures utilized with iron oxide and Si. In this regard, grafted samples were subjected to corrosive acidic (HF or HNO_3_) and basic (cold and hot NaOH) environments. Other procedures, including UV radiation and mechanical cleavage by brushing, were also investigated. However, all of these experiments resulted in inhomogeneous polymer breakage or polymer degradation.

**Figure 1:**
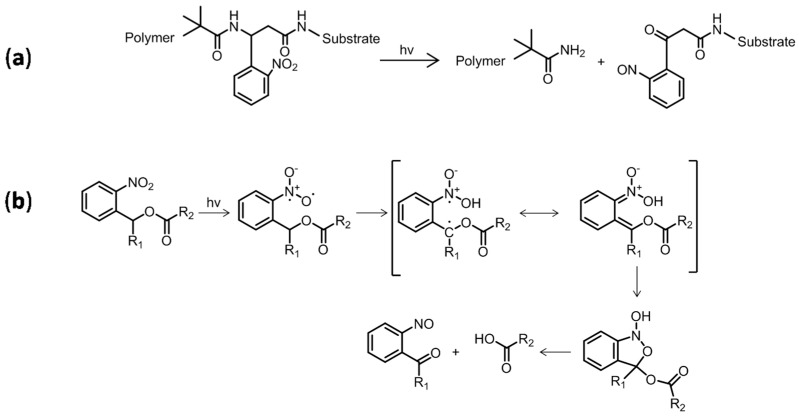
(**a**) Photocleavage of grafted polymer chains by photocleaving substrate *o*−nitrobenzene group [17]; (**b**) proposed process for UV cleavage of *o*-nitrobenzene [19,20].

An innovative technique introduced a photocleavable group between the substrate and polymer chain. Kang et al. removed poly(lauryl methacrylate) and grafted PS chains from silicon substrates using a UV light [17], which allowed selective breakage of the substrate–polymer link (Figure 1a) and, therefore, polymer dissolution. Light-sensitive thiol epoxy networks grafted onto Si were cleaved by Pelliccioli et al. [18]. They subjected samples to UV light below 400 nm to cleave *o*-nitrobenzene bonds through a hydrogen abstraction biradical process. Based on these studies, Romano et al. extended the concept of photocleavage to thiol epoxy systems using *o*-nitrobenzyl ester groups [19]. The authors proposed a photocleavage process as depicted in Figure 1b [20].

**Figure 2:**
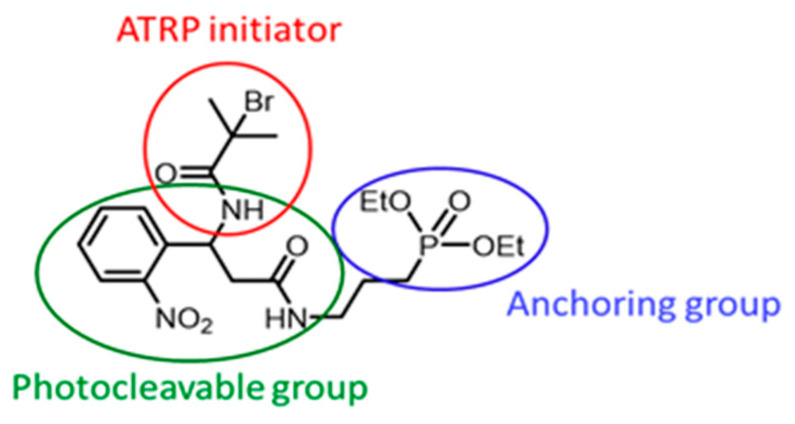
Photocleavable initiator (PCI) chemical structure.

In the current work, a photocleavable SI-ATRP initiator (C_20_H_31_BrN_3_O_7_P, M = 536.35 g·mol^−1^, labelled PCI (Figure 2)) is synthesized and used to grow PMMA chains on Ti. This initiator has three parts: a Ti-anchoring diethyl phosphonate, an *α*-bromo amide ATRP initiator, and a photocleavable *o*-nitrobenzene. A synthesis of the PCI is conducted using a multistep synthesis involving classical organic reactions.

**Figure 3:**
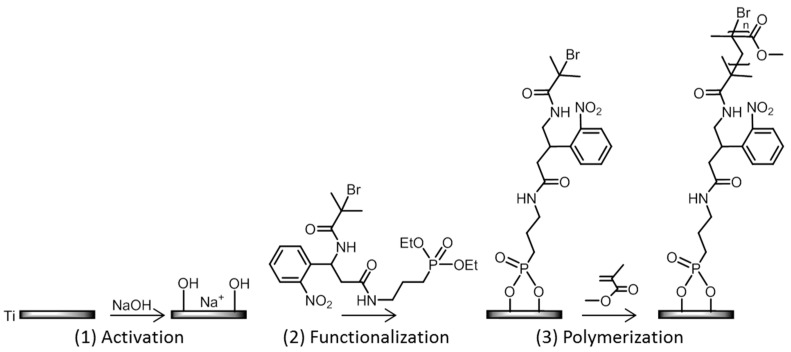
Three-step strategy used to design PMMA-coated Ti surfaces.

This study investigates the validation of the selected photocleavable process and the characterization of PMMA chain growth on Ti plates. The PMMA chains are grown on Ti using a technique previously described by Reggente et al. [16] of replacing the C11 initiator with the PCI. This modification is likely to change the experimental conditions of the functionalization procedure (Figure 3).

## 2. Materials and Methods

### 2.1. Sample Preparation

**Figure 4:**
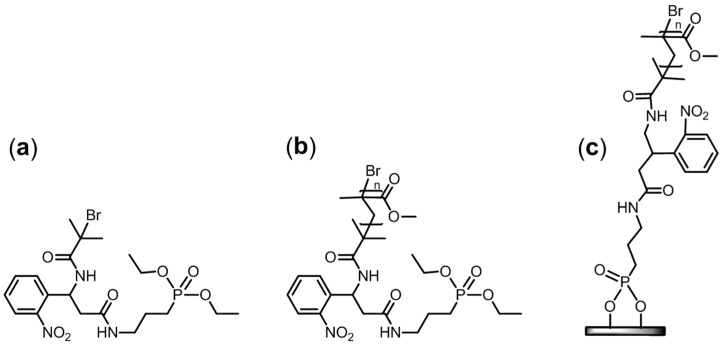
Molecular structures of the three PCI-based materials investigated: (**a**) photocleavable initiator (PCI), (**b**) model PCI-initiated PMMA, and (**c**) PCI-initiated PMMA brushes on Ti.

The study described hereafter concerns the use of a new SI-ATRP initiator with a photocleavable group in order to characterize PMMA grafted chains onto Ti surfaces using ATRP polymerization. This study is divided in three parts (Figure 4):

(i) The synthesis and characterization of the PCI (compound a in Figure 4);
(ii) The validation of the photocleavage process using a model polymer in solution (polymer b in Figure 4);
(iii) The characterization of PMMA chains released from the Ti plates (material c in Figure 4).

#### 2.1.1. Photocleavable Initiator Synthesis

**Figure 5:**
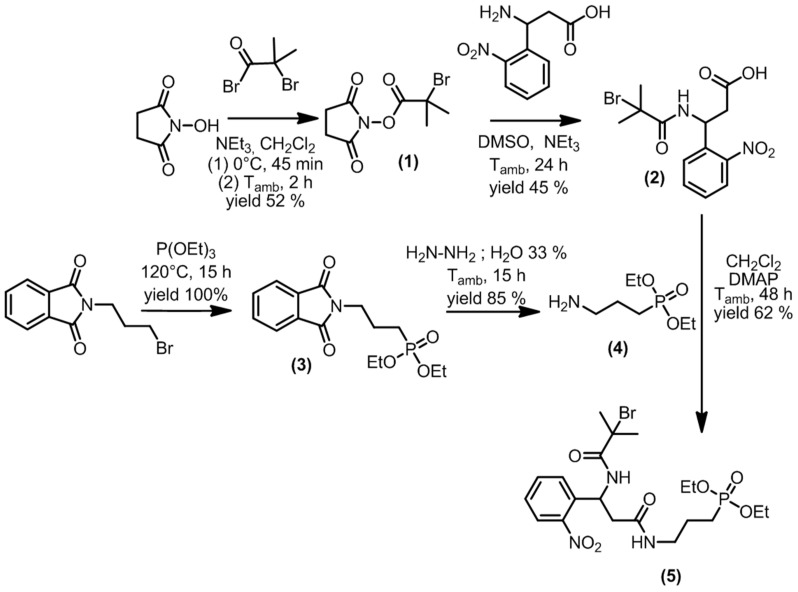
Synthesis procedure of the photocleavable initiator (PCI).

The PCI was synthesized using a five-step strategy, as depicted in Figure 5. First, bromoester **1** was obtained from a reaction between bromoisobutyryl bromide and *N*-hydroxysuccinimide. Bromoester **1** was then subjected to an amidation reaction with 𝛽-amino-2-nitrobenzenepropanoic acid to yield compound **2**. Diethylphosphate **3** was obtained through a Michaelis–Arbuzov reaction between *N*-(3-bromopropyl)phtalimide and triethylphosphite. Subsequent hydrazinolysis led to the formation of amino-diethylphosphate **4**. Finally, an amidation reaction between carboxylic acid **2** and amino-diethylphosphate **4** led to the formation of PCI **5**. All experimental details and characterization data are reported in the supporting information.

#### 2.1.2. Model Polymer Synthesis

**Figure 6:**
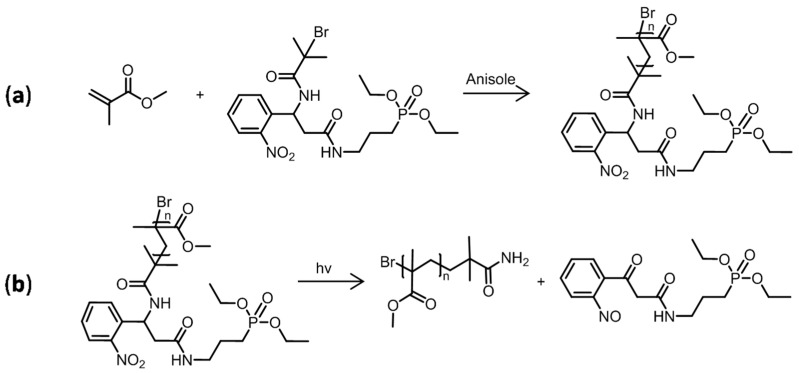
(**a**) Homopolymerization of MMA in solution using the photocleavable initiator; (**b**) probable products resulting from the photocleavage reaction of the grafted PMMA in solution [17].

Before starting the grafting of PMMA onto Ti, it was necessary first to validate the photocleavable ability of the PCI. In order to accomplish this goal, a model polymer in solution was produced by ATRP using MMA in the presence of the PCI and a neat titanium plate (Figure 6a). The chemicals (CuBr as the catalyst, anisole as the solvent, and PMDETA as the ligand) and experimental conditions (T = 35 °C, t = 24 h, and 300 rpm under argon) were identical to those used in the third PMMA-grafting step. Because the goal was to synthesize the polymer in solution rather than grafted onto a surface, the polymerization accelerator malononitrile was not required. To generate a fluid and nonviscous polymer solution, the ratio of [MMA]:[anisole] of 35:65 *v*/*v* was used. After synthesis, the polymer was precipitated in cold MeOH under stirring, filtered, and dried under a vacuum overnight.

The synthesized PMMA in solution was illuminated under a UV lamp (Super High-Pressure Mercury Lamp USH-200DP, Ushio, Tokyo, Japan). This UV lamp presented a large emission spectrum with a maximum at 254 nm. For this, 1 g of PMMA was dissolved in 10 mL of THF in a glass dish and exposed for 30 min at 100 mm from the light source. Then, the solvent was removed and the collected PMMA was dried under a vacuum (Figure 6b).

#### 2.1.3. PMMA Grafting on Ti Plates and Photocleavage

**Sample preparation.** Four titanium plates (20 × 20 × 0.15 mm^3^) were cut from grade-2 Ti. Their surface roughness was 0.5 ± 0.1 μm. Sonication in acetone, ethanol, and deionized water for 10 min was employed to clean the samples. The solvents were purchased from Carlo Erba, Cornaredo, Italy.

As schematized in Figure 3, a three-step strategy was used:

**Alkali-activated Ti surfaces.** Ti samples were activated by immersing them in a 2.0 M sodium hydroxide (NaOH) (Carlo Erba) solution in a Teflon beaker for 1 h at 80 °C while stirring at 300 rpm. A Teflon wire was used to suspend the Ti samples in the reactive solution during the activation process, preventing the possibility of Ti plate contact.

**Initiator-modified Ti surfaces.** The alkali-activated Ti samples were washed with deionized water and then suspended in a Schlenk tube containing a 5.0 mM aqueous initiator (PCI) solution (0.241 g in 90 mL of deionized water) to covalently graft it onto the sample surfaces. The concentration used in this case was twice the C11 concentration used previously [16] because of the weaker reactivity of the phosphonic ester group of the PCI compared to the phosphonic acid of the C11 initiator [21]. The reaction was carried out at 95 °C for 24 h with stirring at 300 rpm under reflux. The grafting procedure was performed in the dark to prevent side reactions caused by the well-known photocatalytic activity of titanium dioxide [22]. After that, the samples were sonicated in dichloromethane and deionized water for 15 min each to eliminate unreacted residual initiator.

**Polymer-coated Ti surfaces.** The initiator-modified Ti samples were moved to a Schlenk tube dedicated only to the polymerization of MMA. The reagents were introduced in the following order under argon:

- First, 40 mg of copper bromide (CuBr) (Sigma Aldrich, St. Louis, MO, USA), 18 mL of anisole (Acros Organics, Waltham, MA, USA), and 50 µL of pentamethylenediethyltriamine (PMDETA) (Acros Organics) were added to the Schlenck tube and agitated to achieve a homogenous solution.
- Second, 20 mg of malononitrile (Acros Organics) was added to the reactive medium to increase the rate of polymerization [23,24]. The solution instantly became black.
- Third, 22 mL of MMA (Acros Organics) filtered from its stabilizer by flowing through a pad of basic aluminum oxide (Acros Organics) was added to the reactor to obtain a 5.3 M monomer solution [16].

Once more, the system was degassed with alternate cycles of vacuum and argon. Under argon, the reaction was conducted for 24 h at 35 °C with stirring at 300 rpm.

At the conclusion of the selected reaction time, the samples were thoroughly washed and sonicated for 10 min in methanol to halt polymerization and remove catalyst residues (such as copper bromide derivatives and PMDETA) and unreacted monomers.

**Collection of PMMA from grafted Ti plates under UV radiation.** Four PMMA-modified Ti samples (20 × 20 mm^2^) were dipped in 10 mL of THF in a glass dish and exposed to a UV lamp for 30 min [15] on each side at 100 mm from the light source (Super High-Pressure Mercury Lamp USH-200DP). The samples were washed with THF at the end of the reaction, the solvent was removed, and the PMMA recovered in solution was concentrated under reduced pressure.

An identical UV light was utilized for both types of photocleavage. THF being transparent above 230 nm, it did not affect the photocleavage process for the model polymer. The same may be said for grafted PMMA, which was UV transparent at the wavelength employed (see Supplementary Materials, Figure S1). Furthermore, it was reported that a dense layer of grafted chains on a substrate promoted photocleavage [17].

## 3. Characterizations

The chemical structures of the PCI (Figure 4, compound a), the PMMA synthesized in solution (Figure 4, compound b), and the polymer-coated Ti surfaces (Figure 4, material c) were analyzed using different techniques, as listed below.

**Infrared spectroscopy**. The reactions were followed by attenuated total reflection Fourier-transform infrared (ATR-FTIR) spectroscopy (PerkinElmer UATR Two, Waltham, MA, USA). The analysis was performed in the 400–4000 cm^−1^ range with eight accumulations.

**Contact angle** (CA). The measures were conducted using a homemade apparatus. A drop of water was deposited on the sample surface in order to determine the wettability of the material. The optical measurements were performed three times for each sample.

**Scanning electron microscopy** (**SEM**) **and energy-dispersive x-ray spectroscopy** (**EDX**)**.** The sample surfaces and cross-section were investigated with SEM (Zeiss GeminiSEM500, Jena, Germany) and EDX (EDAX Octane, Pleasanton, CA, USA). No preparations were made for the surface investigation.

**Cross-section analysis.** The preparation of the cross-sections needed two steps. The first one was tje deposition of a copper (Cu) layer of approximately 25 nm by magnetron sputtering on the sample surface. The second step consisted of producing the cross-sections using an ion beam of argon with a kinetic energy of 6 keV (cross polisher, Hitachi IM4000+, Tokyo, Japan). The PMMA was protected by the Ti itself and exposed to the ion beam in order to obtain the sample cross-sections. Backscattered electrons (BSEs) were measured to capture the images. The Cu layer showed an intense signal compared to the PMMA and allowed the determination of PMMA thickness. A linear analysis was performed to identify the elementary compositions of the different layers.

**Nuclear magnetic resonance spectroscopy.** The ^1^H- and ^13^C-NMR characterizations were performed using deuterated chloroform as the solvent for the synthesized PCI and the collected PMMA chains (Bruker Advance 500 MHz NMR spectrometer, Billerica, MA, USA).

**Size exclusion chromatography.** SEC coupled with a light-scattering analysis (LS-SEC) was performed using a Shimadzu SIL-20A autosampler and a Shimadzu LC20-AD pump (Shimadzu, Kyoto, Japan) fitted with four PLGel mixed C columns (particle size = 5 μm; length = 30 cm; diameter = 7.5 mm; separation range: 1000 to 10 million dalton) in THF using three detection modes: a differential refractometer detector (T-rEX refractometer, Wyatt Technology Corporation, Santa Barbara, CA, USA) calibrated with polymethyl methacrylate standards, a UV detector (Shimadzu SPD-M20A diode array UV detector), and a light-scattering detector (miniDAWN, Wyatt Technology Corporation).

**Mass spectrometry.** Mass spectra were recorded with an Orbitrap Exactive Plus EMR (Thermo Fisher Scientific, Bremen, Germany) mass spectrometer coupled to an automated chip-based nanoelectrospray device (Triversa Nanomate, Advion, Ithaca, NY, USA) operating in positive ion mode at 1.3 kV, and the pressure of the nebulizer gas was 0.40 psi. Using an in-source voltage of 40 eV, ions were moved through the mass spectrometer and thermalized in an HCD cell at 20 eV. The constant nitrogen pressure in the backing source region, the HCD cell, and the orbitrap analyzer were set to 2 × 10^−5^ and 10^−9^ mbar, respectively. External mass calibration was achieved using Tuning Mix (Agilent Technologies, Palo Alto, CA, USA) in the mass range of 322–2732 *m*/*z*.

## 4. Results and Discussion

### 4.1. Photocleavable Initiator

The PCI was prepared in batches of several grams with an overall yield of 12–15%, and its structure was established successfully by ^1^H- and ^13^C-NMR spectroscopies and a mass spectra analysis. The found mass of PCI with Li^+^ was equal to 542.120 g·mol^−1^ for one theoretical value of 542.050 g·mol^−1^. Details of the synthesis and characterization are available in the Supplementary Materials.

### 4.2. Model Polymer

**Figure 7:**
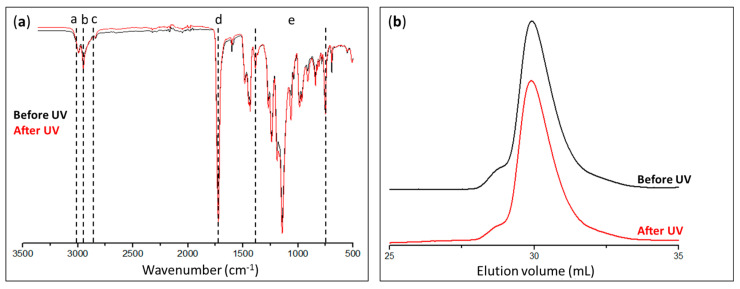
(**a**) Infrared spectra and (**b**) chromatograms of PMMA synthesized in solution before (black curves) and after (red curves) exposure to UV radiation.

The PMMA collected from the photocleavage in the THF solution was characterized by IR spectroscopy (Figure 7a) and SEC (Figure 7b). No differences could be seen in the IR spectra, regardless of whether they were captured before or after exposure to UV radiation, and the same absorption bands are present in both spectra shown in Figure 7a.

Actually, the peaks relative to the PMMA structure could be observed, particularly those in the 2800–3000 cm^−1^ area that corresponded to the –CH_2_– and –CH_3_ bonds, as well as at 1730 cm^−1^, which indicated the existence of a C=O bond.

The amounts of polymers collected were insufficient to perform a ^1^H-NMR chemical structure analysis. The SEC chromatograms of PMMA showed the same pattern both before and after UV exposure, evidencing the absence of significant chain coupling and polymer chain degradation. Additionally, the polymer characterization data gave results of the same magnitude, with values of Mn ≈ 11,000 g·mol^−1^, Mw ≈ 15,000 g·mol^−1^, and Ɖ ≈ 1.35. These outcomes verified the procedure of photocleavage by demonstrating that the recovered polymer did not degrade when exposed to UV light.

### 4.3. Grafted Polymer Characteristics

As the PCI was a novel initiator for surface-initiated polymerization, it was vital to characterize the surfaces and interfaces after its grafting and after the polymerization of MMA. These studies must also be performed **before and after UV radiation** exposure in order to emphasize, if feasible, the influence of this treatment on the physicochemical parameters (FTIR and EDX) and morphologies (CA and SEM) of the samples. The PMMA collected in solution after UV cleavage was then isolated and characterized (FTIR, ^1^H-NMR, and SEC).

**Figure 8:**
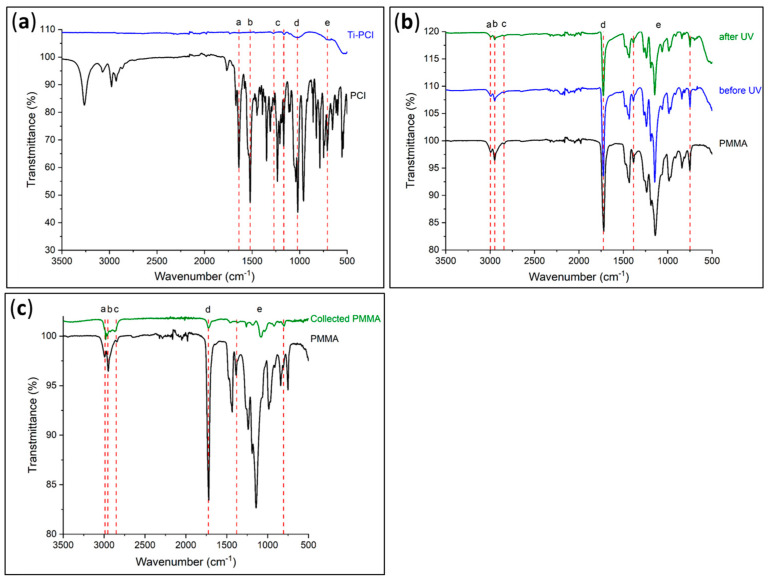
Infrared spectra of (**a**) initiator-modified (Ti–PCI) and (**b**) PMMA-modified (Ti–PMMA) Ti plates before and after exposure to UV radiation, as well as (**c**) collected PMMA under UV radiation.

The effectiveness of the photocleavage was determined using IR spectroscopy; the acquired spectra were compared to the reference spectra of the PCI and PMMA (Figure 8a,c, respectively).

The initial IR spectra in Figure 8a indicated the presence of the PCI on the Ti surface, as evidenced by the different absorption band characteristics of the chemical functional groups in the PCI. It was possible to distinguish the presence of Ti–O–P bonds at 1023 cm^−1^ (d) and the attenuation of P–OCH_2_CH_3_ groups at 709 cm^−1^ (e), which corresponded to a reduction in the number of P–OCH_2_CH_3_ groups, thereby confirming the formation of a bond between the phosphorus atom and the Ti substrate. Other bands could also be observed at 1639 (a) and 1520 cm^−1^ (b), which indicated respectively the existence of C=O groups and the amide function (–NH).

Before being exposed to UV light, the second step consisted of checking the grafting of PMMA onto Ti by examining the absorption bands associated with the polymer (Figure 8b). These included the existence of C=O groups at 1725 cm^−1^ (d), as well as the elongation vibrations of the –CH_2_ and –CH_3_ groups of PMMA in the 2800–3000 cm^−1^ area (a–c). It can be seen from the IR spectra that the polymer was still present on the Ti even after exposure to UV light and that the substance recovered in solution was PMMA (Figure 8c) with the same absorption bands on the surface. Even though the cleavage was not totally effective, 0.14 mg was recovered as opposed to the expected amount of 0.55 mg. This quantity was calculated based on a coating with an average thickness of 120 nm on both sides of four Ti plates of 20 × 20 mm^2^ each. These results are consistent with those of Kang et al. [15], who demonstrated that some polymer was still present on a substrate after exposure to ultraviolet radiation. Indeed, they demonstrated experimentally that the thickness of the polymer on the substrate decreased by 90% after 30 min of UV exposure. In agreement with Kang et al., we also chose 30 min to avoid possible degradation of the PMMA.

A proton-bearing functional group’s existence was discovered using ^1^H-NMR spectroscopy. After photocleavage, the polymer was collected in a THF solution, and its nature was determined by analysis. The resulting spectrum was consistent with that of PMMA, with a peak at a chemical shift of roughly 3.6 ppm specifically confirming the presence of the O–CH_3_ group of PMMA; nevertheless, the signals were quite weak in comparison to the solvent, and no further information could be obtained from NMR spectroscopy.

**Figure 9:**
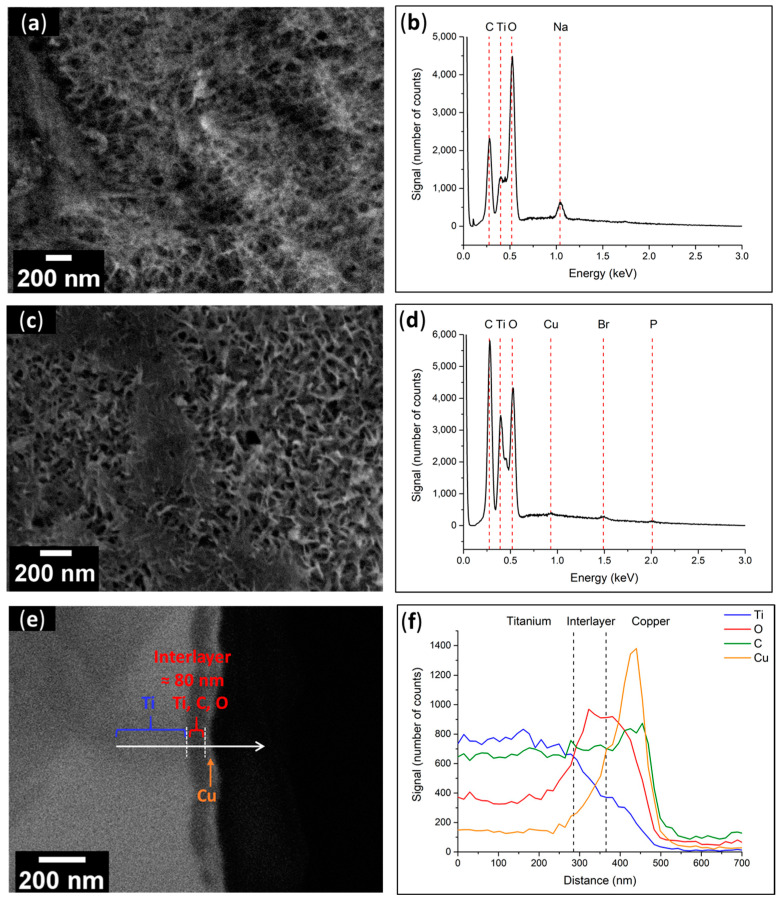
(**a**) SEM top view image and (**b**) EDX spectrum of the Ti–OH surface; (**c**) SEM top view image and (**d**) EDX spectrum of the Ti–PCI surface; (**e**) SEM cross-sectional view and (**f**) Linear analysis of the Ti–PCI interface.

Below are presented and discussed the SEM pictures, the CA values, and the EDX studies of the initiator-grafting (Ti-PCI) and MMA polymerization stages before and after exposure to UV light. The activation step allowed the generation of active sites on the metal surfaces (Ti–OH) to anchor the initiator with its diethyl phosphonic group. This layer consisted of a porous and hydrophilic structure (CA = 21 ± 3°) due to the presence of hydroxyl groups at the surface (Figure 9a) [25]. Figure 9c demonstrates that the grafting reaction (Ti-PCI) resulted in a reorganization of the surface morphology accompanied by the creation of an organic layer. This was evidenced by a reduction in the surface wettability of Ti-PCI (CA = 60 ± 2°) compared to the hydroxylated surface (CA = 21 ± 3°). It can be also observed that the Ti-PCI remained hydrophilic because of the presence of polar nitro (–NO_2_) and amide (NH–CO) groups in its chemical structure.

The global EDX spectrum of the activated surfaces (Figure 9b) showed the presence of Ti, O, and Na, which confirmed the presence of the sodium titanate layer. Figure 9d demonstrates the presence of C and O and the absence of Na, which indicates that the initiator covered the porous layer of sodium titanate, as well as P and Br, which are chemical elements corresponding to the diethyl phosphonic (P–(OEt)_2_) and *α*-bromo amide (NHCOC(CH_3_)_2_Br) moieties of the initiator. On this spectrum, the signal related to the N atom merged with that corresponding to Ti, rendering it impossible to discern. Three zones can clearly be seen in the SEM cross-section image (Figure 9e): from left to right, the Ti plate, an interlayer with a thickness of about 80 nm, and the previously deposited Cu layer. The linear analysis performed by EDX spectroscopy (Figure 9f) provided confirmation about the chemical elements present in the various layers. Indeed, there was a left-to-right drop in the Ti signal (blue), increases in the O and C signals (red and green, respectively), and the presence of a Cu layer on the far right (orange).

**Figure 10:**
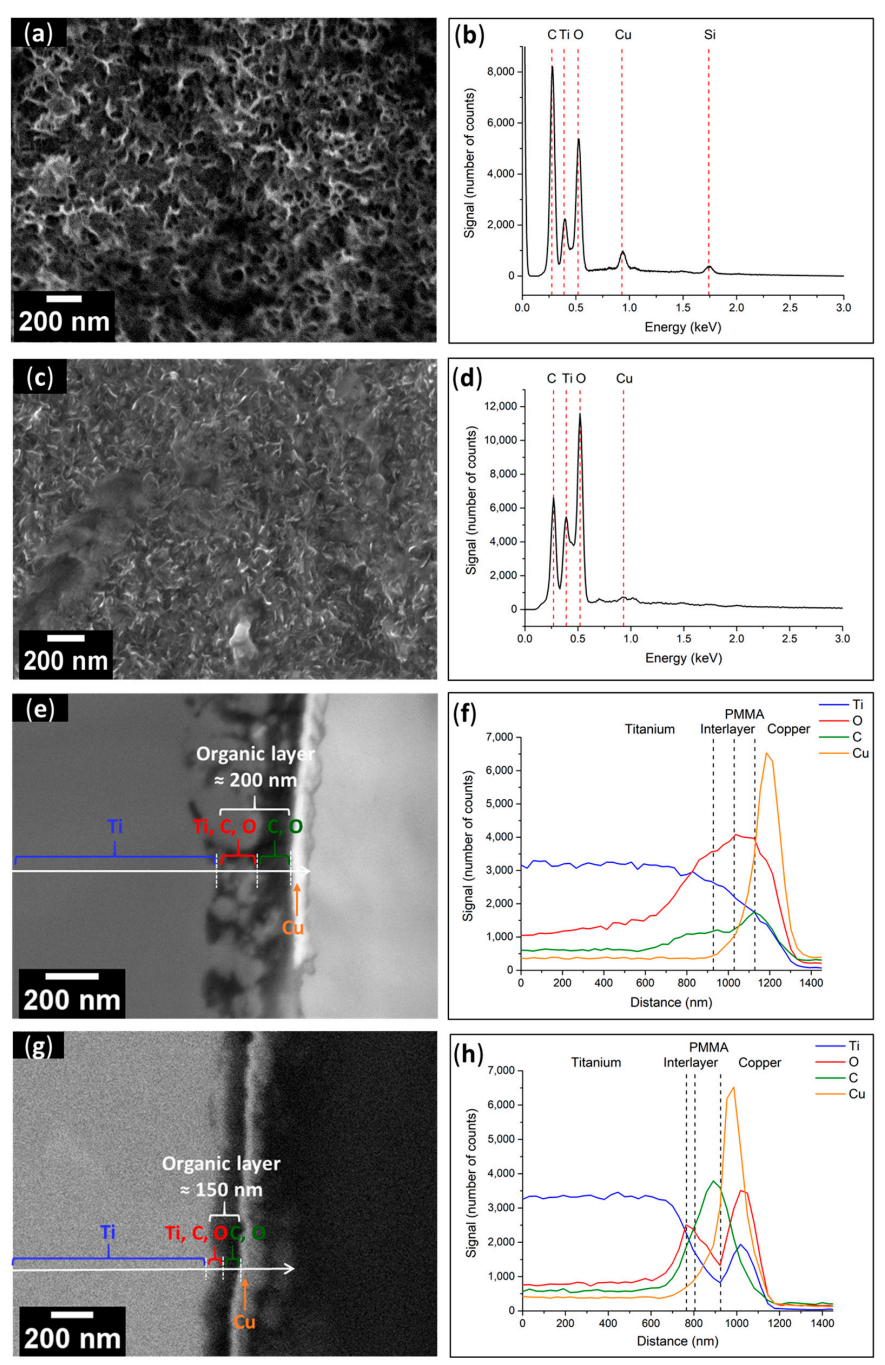
(**a**) SEM top-view image of Ti–PMMA and (**b**) EDX spectrum of Ti–PMMA surface before UV exposure; (**c**) SEM top-view image and (**d**) EDX spectrum of Ti-PMMA surface after UV exposure; (**e**) SEM cross-section and (**f**) linear analysis of Ti–PMMA interface before UV exposure; (**g**) SEM cross-section and (**h**) linear analysis of Ti–PMMA interface after UV exposure.

Figure 10a depicts the polymer as a uniformly dispersed porous layer throughout the full surface of Ti-PMMA before UV exposure. The morphological features were similar to those previously reported, resulting in no change in surface wettability (CA = 59 ± 1°). In addition, the global EDX surface analysis (Figure 10b) indicated the presence of C and O atoms and the absence of P and Br, which correlated to the existence of polymers on the surface. As illustrated in Figure 10c, *exposure to UV light* did not alter the appearance of the PMMA surface morphology. In addition, the surface wettability was of the same magnitude as before (CA = 61 ± 3° after UV exposure). In addition, the EDX examination of the surface (Figure 10d) revealed the existence of C and O, with a lower intensity for C; hence, it can be inferred that PMMA was still present on the Ti plate.

These findings were supported by the cross-sectional views and linear EDX spectroscopy investigations, which showed similar results before and after UV light exposure. Furthermore, the thickness of the organic layer was reduced following UV exposure (≈200 nm before and ≈150 nm after). To illustrate this, SEM pictures of the sections and linear spectroscopic EDX analyses of the Ti-PMMA plates before (Figure 10e,f) and after (Figure 10g,h) UV radiation treatment are displayed. For both before and after UV exposure, four layers were observed, including from left to right, (i) the Ti substrate; (ii) an interlayer containing Ti, O, and C recovered from (iii) the polymer layer where C and O signals were at their highest intensities; and finally, the previously deposited Cu layer.

The thicknesses were estimated using cross sectional views in SEM. Specifically, Figure 9e for Ti-PCI, Figure 10e for Ti-PMMA before UV exposure, and Figure 10g after UV exposure were 270 ± 80 nm before and 200 ± 50 nm after UV exposure, respectively. Each sample received three measurements, which were noted as “means ± standard deviation”. These observations were performed on additional samples from a second experimentation to ensure that the process was reproducible. We could observe a comparable effect of decrease in the organic layer thickness after UV exposure.

### 4.4. PMMA in Solution

Despite the very small amount of PMMA recovered, an SEC analysis could be performed that clearly evidenced the presence of photocleaved polymers.

**Figure 11:**
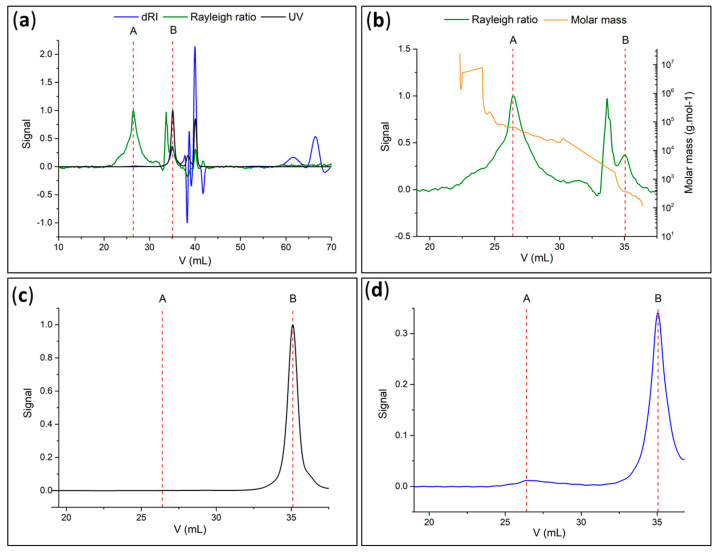
(**a**) Chromatogram of photocleaved PMMA recovered in solution (green: Rayleigh ratio or light-scattering signal; blue: refractometer signal; black: UV at 254 nm); (**b**) molar mass (green: light-scattering signal; orange: molar mass); (**c**) signal in UV at 254 nm and (**d**) refractometer signal of photocleaved PMMA recovered in solution.

Actually, PMMA corresponded to peak A observed at 26.4 mL (low elution volumes, high molar masses), whereas peak B detected during UV exposure was at 35.1 mL (high elution volumes) (Figure 11a). For peak A, the determined values of Mn and Mw from the refractometer signal and Rayleigh ratio of the SEC (Figure 11b,d) were 40,000 g·mol^−1^ and 51,000 g·mol^−1^, respectively, and Ɖ was 1.28. These experimental values are comparable to the experimental values obtained by Kang et al. (Mn ≈ 50,000 g·mol^−1^, Mw ≈ 80,000 g·mol^−1^, and Ɖ ≈ 1.60) for cleaved PS chains from silica surfaces previously obtained by SI-ATRP [17].

Considering the degree of polymerization (DPn = 400, derived from SEC data), the polymer chain length for the case of fully stretched chains was estimated to be 101 nm [26]. This value is of the same order of magnitude as the 120 nm thickness of PMMA determined by SEM.

Due to steric restrictions [27] during the polymerization process, the molecular masses of a grafted polymer on metal probably cannot reach high values. It is also important to note that the Mn molecular value is higher than the entanglement mass of PMMA (Me ≈ 10,000–11,000) [28], which allows good cohesion and mechanical properties of sandwich materials (Ti/PMMA/Ti) prepared by the alternative superposition of functionalized Ti plates and massive PMMA layers [29]. The entanglement phenomenon between PMMA chains covalently linked to Ti plates and the chains of a massive layer avoids delamination between the constituents of sandwich materials [29].

Peak B at high elution volumes or low masses (Figure 11c,d) corresponded to small molecules, such as PCI fragments formed from the degradation of the PCI by UV irradiation. Moreover, the UV signal (Figure 11c) indicated the presence of absorbing molecules, likely aromatics, such as an initiator component [17]. Additionally, it was predicted that UV radiation could release the polymer chains by breaking up the PCI linker, resulting in low-molar-mass residues. In conclusion, the SEC analysis validated the collection of PMMA and found agreement with the IR and ^1^H-NMR spectra.

A second round of measurements was carried out on additional samples to verify the reproducibility of the photocleavage process and the characteristics of the recovered polymers. The SEC results confirmed the presence of one peak at low elution volumes, indicating the presence of PMMA at similar values, i.e., Mn = 35,000 g·mol^−1^, Mw = 40,000 g·mol^−1^, and Ɖ = 1.14.

## 5. Conclusions

The objective of this study was to characterize grafted PMMA on a Ti plate after cleavage of the strong bonds between Ti and the PMMA chains. Initial procedures using chemical reagents and exposure to UV radiation or mechanical brushing on Ti-PMMA surfaces prepared using the “grafting-from” procedure (i.e., by surface-initiated atom transfer polymerization (SI-ATRP)) developed by Reggente et al. [16] resulted in inconsistent cleavage or degradation of the polymer. Using the same polymer-grafting technique, a new initiator containing a photocleavable *o*-nitrobenzene group was prepared and employed to graft PMMA onto the Ti plates. The presence of the photocleavable o-nitrobenzene group allowed the extraction of PMMA from the Ti substrate using UV light. This new process was validated firstly on a model polymer in solution and led to no evidence of PMMA degradation. This constitutes a novel and valuable method to recover under mild conditions a polymer grafted on any substrate in order to undertake its detailed characterization in solution, in particular.

Despite steric constraints involved in such a surface-initiated polymerization procedure, the Mn and Mw values of PMMA brushes collected from Ti plates were in the tens of thousands, and the Ɖ was quite low, indicating that polymer chains grew efficiently and fairly uniformly. It may, therefore, be assumed that this surface-initiated (ATRP) polymerization proceeded in a similar way to classical, controlled radical polymerization in solution.

Moreover, the Mn values of the PMMA brushes were significantly higher than the entanglement mass of PMMA, permitting good mechanical properties of the Ti/PMMA/Ti sandwich materials. The IR spectra revealed after UV treatment the presence of residual PMMA on Ti, which indicated partial photocleavage and, therefore, partial cleavage of metal–polymer bonds. Before and after UV irradiation, morphological analyses of Ti surfaces and interfaces revealed a thinner organic layer of roughly 200–250 nm. Although only small amounts of grafted PMMA in solution were obtained, ^1^H-NMR, IR, and SEC analyses confirmed the nature of the polymer chains formed.

## Data Availability

Not applicable.

## Supplementary Materials

The following supporting information can be downloaded at: https://www.mdpi.com/article/10.3390/polym15051265/s1, Figure S1: Absorption spectra of PMMA and of PCI initiator in dichloromethane solution (5 × 10^−4^ M). At the wavelengths of the mercury lamp used for the photocleavage, only the PCI unit is absorbing.
