# Supplementary material for: Use of a Photocleavable Initiator to Characterize Polymer Chains Grafted onto a Metal Plate with the Grafting-from Method
Source: Polymers (Basel). 2023 Mar 2;15(5):1265. doi: 10.3390/polym15051265 (PMC10007346; doi:10.3390/polym15051265)
Supplement: Supplementary file 1 [file polymers-15-01265-s001.zip › polymers-2205677-supplementary.pdf]

Supplementary Material

Synthesis and characterization of PCI

^{1}H-NMR and ^{13}C-NMR (500 MHz and 125 MHz respectively) spectra were recorded with a Bruker Ascend 500 spectrometer at 25 °C. Chemical shifts, *δ*, are reported in ppm using the solvent ($CDCl_3$ or DMSO-d_6) as internal standard (7.26 or 2.54 ppm, respectively); spin-spin coupling constants, *J*, are given in Hz and the abbreviations s, br, t, q, m were used to denote respectively the multiplicity of signals: singlet, broad singlet, triplet, quadruplet, multiplet.

Compound 1. To a stirred solution of *N*-Hydroxysuccinimide (9.2 g, 80 mmol, 1 eq), dry triethylamine (22.2 mL, 160 mmol, 2 eq) and dry dichloromethane (150 mL) was added dropwise bromoisobutyryl bromide (11.1 mL, 88 mmol, 1.1 eq) at 0 °C. The resulting mixture was stirred for 45 min and allowed to warm up to room temperature for another 2 h. It was then poured into cold water and extracted with diethyl ether (2 x 50 mL). Organics were washed successively with saturated Na_2CO_3, 10 % HCl and saturated Na_2CO_3 again. Organics were dried with anhydrous $MgSO_4$, filtered and concentrated using a rotary evaporator to give an off-white solid (14.3 g) with 68 % yield. ^{1}H-NMR ($CDCl_3$) *δ* 2.86 (s, 4H, -CH_2), 2.08 (s, 6H, -CH_3).

Compound 2. 3-Amino-3-(2-nitrophenyl)propionic acid (3.5 g, 16 mmol) and compound **1** (5.2 g, 20 mmol, 1.2 eq) were dissolved in DMSO (50 mL) in a 250 mL round bottomed flask. Triethylamine (6 mL, 43 mmol, 2.7 eq) was added with an addition funnel over a period of 15 min. The reaction mixture was stirred at room temperature for 30 h. DMSO was evaporated under vacuum and water (100 mL) was added. The aqueous phase was extracted with AcOEt (90 mL), then acidified with concentrated HCl (pH = 2), and finally extracted again with AcOEt (3 x 60 mL). Organics were combined and washed with 1 M HCl (60 mL), H_2O (80 mL), dried over Na_2SO_4 and concentrated under reduced pressure to give 2.5 g of a light brown solid with 45 % yield. ^{1}H-NMR (DMSO-d6) *δ* 8.74 (d, *J* = 7.35 Hz, 1H, -NH), 7.92 (d, *J* = 7.92 Hz, 1H, HAr), 7.72 (d, *J* = 4.62 Hz, 2H, HAr), 7.52-7.47 (m, 1H, HAr), 5.56-5.51 (m, 1H, C*H), 2.93-2.73 (m, 2H, -CH_2), 1.83 (d, *J* = 12.06 Hz, 6H, -CH_3).

Compound 3. *N*-(3-Bromopropyl)phthalimide (10 g, 37 mmol, 1 eq) and triethylphosphite (40 mL, 222 mmol, 6 eq) were refluxed at 180 °C overnight. Remaining triethylphosphite was removed with reduced pressure to give 12.7 g of a light yellow oil with quantitative yield. ^{1}H-NMR ($CDCl_3$) *δ* 7.86-7.83 (m, 2H, HAr), 7.73-7.70 (m, 2H, HAr), 4.11-4.03 (m, 4H, -C*H*$_2$CH$_3$), 3.75 (t, *J* = 6.93 Hz, 2H, -CH_2N), 1.97-1.94 (m, 2H, -CH$_2$C*H*$_2$P), 1.83-1.77 (m, 2H, -C*H*$_2$CH$_2$P), 1.30 (t, *J* = 7.14 Hz, 6H, -CH_3).

Compound 4. Compound **3** (1 g, 3 mmol, 1 eq) was dissolved in EtOH (100 mL). Hydrazine 33 % solution in water (5.4 mL, 20 eq) was prepared by mixing 2.7 mL of 65 % hydrazin hydrate with 2.7 mL of distilled water, and was added dropwise to the mixture. The reaction was allowed to stir overnight at room temperature. The white resulting precipitate was filtered off and washed with cold ethanol (50 mL). The filtrate was concentrated and dichloromethane was added (100 mL). The organic phase was washed with saturated NaCl, dried, filtered over Na_2SO_4 and concentrated to give a yellowish oil (0.5 g, 2.5 mmol) with 81 % yield. ^{1}H-NMR ($CDCl_3$) *δ* 4.14-4.05 (m, 4H, -C*H*$_2$CH$_3$), 2.76 (t, *J* = 6.78 Hz, 2H, CH_2N), 1.79-1.71 (m, 4H, 2x-CH_2), 1.32 (t, *J* = 7.14 Hz, 6H, -CH_3).

Compound 5. This reaction was conducted in dry conditions and under argon atmosphere.Compound **2** (1.7 g, 5.12 mmol, 1 eq), compound **4** (1 g, 5.12 mmol, 1 eq) and dimethylaminopyridine (0.31 g, 2.6 mmol, 0.5 eq) were dissolved in 100 mL dry dichloromethane. Ethyl-3-(3-dimethylaminopropyl) carbodiimide (EDC) (1 mL, 5.6 mmol, 1,1 eq) was added dropwise and the mixture was allowed to stir at room temperature over 48 h. The reaction mixture was washed with saturated NaCl solution. Organics were dried

over Na_2SO_4, filtered and concentrated under vacuum. The crude was purified by column chromatography on silica gel using DCM/MeOH 9:1 as eluent. Yellow oil (1.6 g) was recovered with 62 % yield. ^{1}H-NMR ($CDCl_3$) δ 9.15 (d, J = 6.8 Hz, 1H, C*-NH), 7.83 (d, J = 8.2 Hz, 1H, HAr), 7.50 (d, J = 7.9 Hz, 1H, HAr), 7.44 (t, J = 7.5 Hz, 1H, HAr), 7.26 (t, J = 8.4 Hz, 1H, HAr), 6.90 (br, 1H, CH_2-NH), 5.56 (q, J = 5.05 Hz, 1H, -C*H), 3.89 (m, 4H, -C$H_2$$CH_3$), 3.07 (q, J = 6.4Hz, 2H, CH_2-NH), 2.7 (qd, 2H, CH_2-C*), 1.82-1.74 (d, 6H, CH_3-CBr), 1.54-1.37 (m, 4H, C$H_2$$CH_2$-P), 1.17-1.13 (m, 6H, C$H_3$$CH_2$). ^{13}C NMR ($CDCl_3$) δ 171.41, 170.89, 148.18, 136.92, 133.61, 128.36, 124.91, 61.80, 60.67, 48.02, 39.47, 32.09, 31.79, 23.53, 22.19, 16.47. Mass Spectrometry-ElectroSpray(ESI) m/z calcd for $[M+Li]^+$ 542.050, found 542.120

UV spectrum of PCI and PMMA

UV spectra of PCI and PMMA in dichloromethane are presented in **Figure S1**. The spectra clearly show the transparency of the solution of PMMA (5×10^{-4} M) between 240 and 500 nm, while PCI absorbs (5×10^{-4} M, $\varepsilon \approx 1000$ L.mol^{-1}.cm^{-1}) in the UV region up 390 nm. Note that the same UV lamp was used for the two photocleavages.

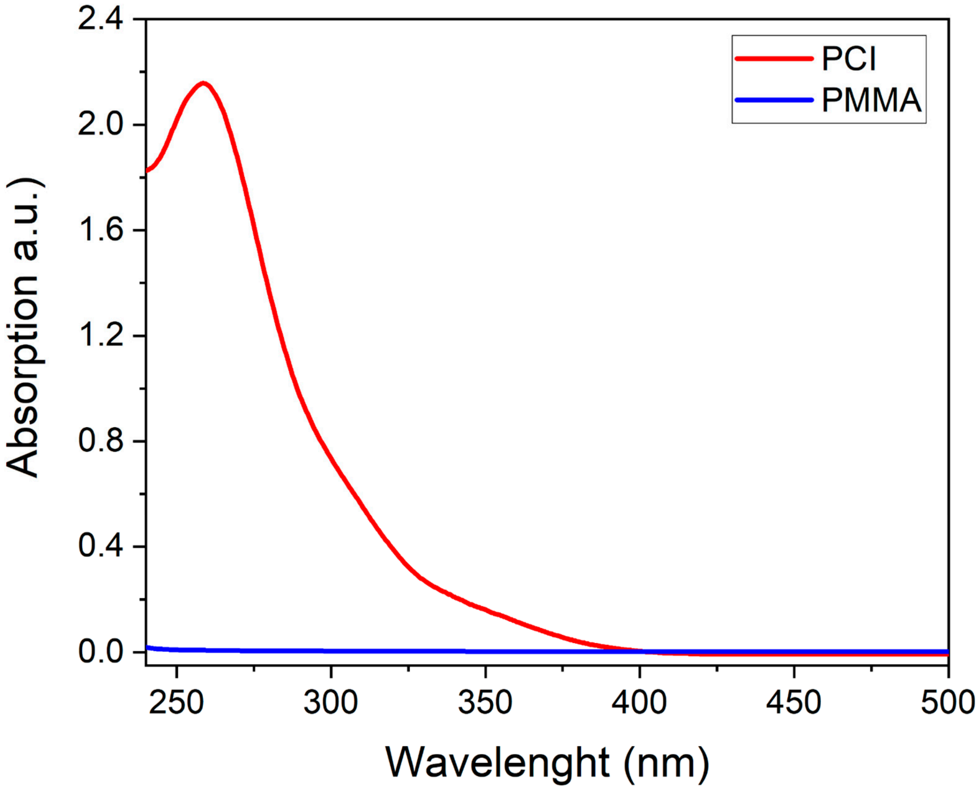

Figure S1. Absorption spectra of PMMA and of PCI initiator in dichloromethane solution (5×10^{-4} M). At the wavelengths of the mercury lamp used for the photocleavage, only the PCI unit is absorbing.
